# edgeR: a versatile tool for the analysis of shRNA-seq and CRISPR-Cas9 genetic screens

**DOI:** 10.12688/f1000research.3928.2

**Published:** 2014-10-21

**Authors:** Zhiyin Dai, Julie M. Sheridan, Linden J. Gearing, Darcy L. Moore, Shian Su, Sam Wormald, Stephen Wilcox, Liam O'Connor, Ross A. Dickins, Marnie E. Blewitt, Matthew E. Ritchie

**Affiliations:** 1 Molecular Medicine Division, The Walter and Eliza Hall Institute of Medical Research, Parkville, Victoria, 3052, Australia; 2 Stem Cells and Cancer Division, The Walter and Eliza Hall Institute of Medical Research, Parkville, Victoria, 3052, Australia; 3 Department of Medical Biology, The University of Melbourne, Parkville, Victoria, 3010, Australia; 4 Systems Biology and Personalised Medicine Division, The Walter and Eliza Hall Institute of Medical Research, Parkville, Victoria, 3052, Australia

## Abstract

Pooled library sequencing screens that perturb gene function in a high-throughput manner are becoming increasingly popular in functional genomics research. Irrespective of the mechanism by which loss of function is achieved, via either RNA interference using short hairpin RNAs (shRNAs) or genetic mutation using single guide RNAs (sgRNAs) with the CRISPR-Cas9 system, there is a need to establish optimal analysis tools to handle such data. Our open-source processing pipeline in edgeR provides a complete analysis solution for screen data, that begins with the raw sequence reads and ends with a ranked list of candidate genes for downstream biological validation. We first summarize the raw data contained in a fastq file into a matrix of counts (samples in the columns, genes in the rows) with options for allowing mismatches and small shifts in sequence position. Diagnostic plots, normalization and differential representation analysis can then be performed using established methods to prioritize results in a statistically rigorous way, with the choice of either the classic exact testing methodology or generalized linear modeling that can handle complex experimental designs. A detailed users’ guide that demonstrates how to analyze screen data in edgeR along with a point-and-click implementation of this workflow in Galaxy are also provided. The edgeR package is freely available from http://www.bioconductor.org.

## Introduction

Pooled library sequencing screens couple gene knock-down/editing technology with second generation sequencing to allow researchers to elucidate gene function in an unbiased, high-throughput manner ^1, 2^. Several recent high impact studies have exploited this approach to discover novel genes involved in processes including cell fate decisions of normal and cancer cells, drug resistance, and to generate genetic interaction maps in mammalian cells using RNA interference (RNAi) ^3– 5^ and sgRNAs with the clustered regularly interspaced palindromic repeats-Cas9 (CRISPR-Cas9) genome editing system ^2, 6^.

Pooled screening relies on the stable genomic integration (often by viral transduction) of a library of uniquely identifiable expression constructs within a population of cells. Each construct expresses an RNA transcript that targets nuclease machinery to a specific nucleotide sequence. This is currently achieved in two main ways: shRNAs can be designed to target specific mRNA transcripts for degradation via the DICER/RISC pathway ^7^ or sgRNAs can be designed to target a co-expressed Cas9 nuclease to a specific sequence in the genome ^8^. By targeting constitutive exons at the 5’ region of a gene, Cas9-mediated double-stranded breaks are repeatedly repaired by nonhomologous end joining until a mutation is introduced that renders the site unrecognisable by the sgRNA. Such mutations typically comprise an insertion or deletion and can give rise to altered coding sequences, disrupted splice sites, frame shifts and/or premature stop codons in the target gene ^8^.

**Figure 1.**
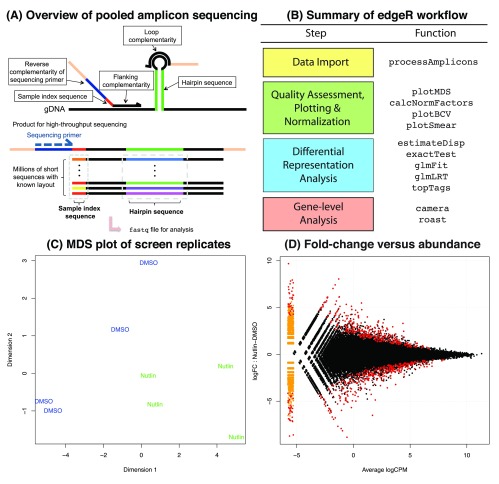
Summary of the raw data, workflow and diagnostic plots from edgeR. ( **A**) Structure of the amplicons sequenced in a typical shRNA-seq screen. Each amplicon will contain sample and hairpin specific sequences at predetermined locations. In sgRNA-seq screens, the amplicon sequences have a similar structure, with the sgRNA sequence replacing the hairpin. After sequencing, the raw data is available in a fastq file. ( **B**) The main steps and functions used in an analysis of shRNA/sgRNA-seq screen data in edgeR are shown. ( **C**) Example of a multidimensional scaling (MDS) plot showing the relationships between replicate dimethyl sulfoxide (DMSO) and Nutlin treated samples (data from Sullivan *et al.* (2012) ^4^). MDS plots provide a quick display of overall variability in the screen and can highlight inconsistent samples. ( **D**) Plot of log _2_-fold-change versus hairpin abundance (log _2_CPM) for the same data. Hairpins with a false discovery rate < 0.05 from an exact test analysis in edgeR (highlighted in red) may be prioritized for further validation.

Depending on the biological question of interest, typically two or more cell populations are compared either in the presence or absence of a selective pressure, or as a time-course before and after a selective pressure is applied. Gain of shRNA/sgRNA representation within a pool suggests that disrupting target gene function confers some sort of advantage to a cell. Similarly, genes whose knockdown/knockout is disadvantageous may be identified through loss of shRNA/sgRNA representation. Screening requires a library of constructs in a lentiviral or retroviral vector backbone that is used to generate a pool of virus for transducing cells of interest. The relative abundance of these constructs in transduced cells is then quantified by PCR amplification of proviral integrants from genomic DNA using primers designed to amplify all cassettes (shRNA/sgRNA) equally, followed by second-generation amplicon sequencing ( Figure 1A). Sample-specific primer indexing allows many different conditions to be analyzed in parallel.

As the popularity of these approaches grows, there is a need to develop suitable analysis pipelines to handle the large volumes of raw data that each screen generates. The major steps in an analysis involve processing the raw sequence reads, assessing the data quality and determining representational differences in the screen in a statistically rigorous way.

Two pipelines are currently available for this task that have been tailored for data from shRNA-seq screens. The shALIGN program ^9^ is a custom Perl script that trims the sequence reads to the pre-defined base positions and then matches these to a library of hairpin sequences. Mismatch bases are permitted, and any ambiguous matches are ignored from the final hairpin count. Statistical analysis of the data is then performed using the shRNAseq R package ^9^, which calculates log-ratios of the counts from each screen replicate, normalizes these values and ranks hairpins by their median, mean or *t*-statistic. Another solution is the BiNGS!SL-seq program ^10^ that uses Bowtie to perform sequence mapping followed by statistical analysis in edgeR ^11^.

In this article, we describe a complete analysis solution for shRNA/sgRNA-seq screens accessible from within the edgeR package available from Bioconductor ^12^.

## Implementation

A summary of the main steps in a typical shRNA/sgRNA-seq analysis alongside the functions in edgeR that perform each task is given in Figure 1B.

### Sequence pre-processing

Our sequence counting procedure has been tailored for screens where PCR amplified shRNA/sgRNA constructs of known structure are sequenced using second generation sequencing technology ( Figure 1A). The location of each index and hairpin/guide sequence is used to determine matches between a specified list of index and hairpin/guide sequences expected in the screen with the sequences in the fastq file. Mismatches in the hairpin/guide sequence are allowed to accommodate sequencing errors, as are small shifts in the position of these sequences within the read. Analysis of unpublished in-house data reveals that allowing for mismatches can yield up to 4.4% additional reads, and shifting an extra 2.6%. This simple searching strategy is implemented in C, with the user interface provided by the processAmplicons function in edgeR. Input to this function consists of a fastq file/s, a second file containing sample IDs and their index sequences and a third file listing hairpin/guide IDs and their respective sequences (the latter files are tab-delimited). A screen with 100 million reads (one lane from an Illumina HiSeq 2000) can be processed in 2–15 minutes depending on the processing parameters. Fastq processing requires minimal RAM, allowing analysis to be completed on any standard computer with R ^13^ installed.

The matrix of counts returned by the processAmplicons function, which contains genes in the rows and samples in the columns, is stored as a DGEList object so that it is fully interoperable with the downstream analysis options available in edgeR. Such an object can also be created directly by the user in the event that these counts have been summarized by alternate means.

Next, the data quality of a screen can be assessed conveniently using multidimensional scaling (MDS) plots via plotMDS ( Figure 1C) and access to a range of normalization options is available through the calcNormFactors function.

### Differential representation analysis

The shRNAseq software ^9^ assumes simple experimental set-ups (e.g. comparing two conditions) that are unsuitable in more complicated situations, such as time-course designs. In edgeR, screens can be analyzed using either the classic method ^14^, ideal for simple two-group comparisons, or generalized linear models (GLMs) ^15^ for more complex screens with multiple conditions (using the glmFit function). This framework can accommodate hairpin/guide-specific variation of both a technical and biological nature as estimated via the estimateDisp function and visualized using plotBCV, which plots biological variability as a function of average hairpin/guide abundance. Robust regression is also possible via the use of observation weights that are estimated using the estimateGLMRobustDisp function ^16^. Statistical testing for changes in shRNA/sgRNA abundance between conditions of interest (typically over time) is carried out using exact (see exactTest function) or likelihood ratio ( glmLRT) tests that allow results to be ranked by significance using the topTags function and plotted using the plotSmear function ( Figure 1D).

Gene set analysis tools available via roast ^17^ and camera ^18^ allow researchers to further test and prioritize screen results. This capability can be used to obtain a gene-by-gene ranking, rather than a hairpin/guide-specific one, which can be helpful when shRNA or sgRNA libraries contain multiple hairpins or guides targeting each gene.

### Case studies and further extensions

**Figure 2.**
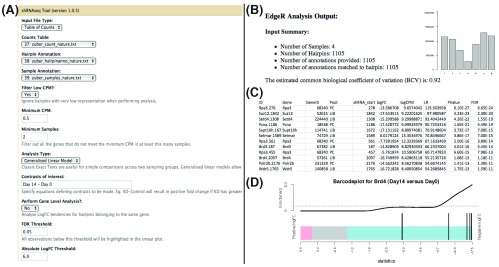
Screenshots of the Galaxy tool for analyzing pooled genetic sequencing screens using edgeR. ( **A**) From the main screen, the user selects the appropriate input files and analysis options. ( **B**) The results of an analysis are summarized in an HTML page that includes various diagnostic plots. ( **C**) Output also includes a table of ranked results at the hairpin/guide and gene-level (where appropriate) as well as barcode plots ( **D**) that highlight the ranks of hairpins/guides targeting a specific gene relative to all other hairpins/guides in the data set.

We provide example data sets and a complete analysis script that demonstrate how to use the edgeR package to prioritize data from four different shRNA-seq screens and two sgRNA-seq screens ^19^. These examples were chosen to showcase edgeR’s ability to deal with experiments of varying size (from tens to thousands of genes) and complexity, from two-group situations, to settings with four groups, or a time-course design, where a GLM with a slope and intercept term is most appropriate. We have also developed a Galaxy tool ^20– 22^ that implements this workflow as a point-and-click application to improve accessibility for researchers who are unfamiliar with the R programming environment ( Figure 2).

## Discussion

Although the major functionality of edgeR has been developed with RNA-seq data in mind, the analysis of numerous in-house data sets ^19^ and the results of others ^4^ have demonstrated its utility for count data derived from pooled amplicon sequencing screens. edgeR provides users with a unique tool for the analysis of data from this emerging application of second generation sequencing technology, that is capable of handling both the biological variability and experimental complexity inherent in these screens. Provision of a Galaxy module puts these powerful statistical methods within reach of experimentalists. Future work will be focused on the use of a suitable control data set to compare this analysis pipeline with other approaches such as shRNAseq ^9^.

## Software availability

### Software access

The edgeR software is an R ^13^ package distributed as part of the Bioconductor project ^12^ ( http://www.bioconductor.org). The Galaxy tool that implements this workflow is available from http://toolshed.g2.bx.psu.edu/view/shians/shrnaseq.

### Latest source code

http://www.bioconductor.org/packages/release/bioc/html/edgeR.html

### Archived source code as at the time of publication

http://dx.doi.org/10.5281/zenodo.12267 ^23^

### Software license

GNU GPL version 2.
