# Supplementary material for: edgeR: a versatile tool for the analysis of shRNA-seq and CRISPR-Cas9 genetic screens
Source: F1000Res. 2014 Oct 21;3:95. Originally published 2014 Apr 24. [Version 2] doi: 10.12688/f1000research.3928.2 (PMC4023662; doi:10.12688/f1000research.3928.2)
Supplement: Peer review history

# Peer review: Referee response for version 2

**DOI:** 10.5256/f1000research.5936.r6477

**Published:** 2014-10-21

**Authors:** Sumit Deswal

**Affiliations:** 1 Department of Molecular Immunology, The Research Institute of Molecular Pathology, Vienna, Austria

The authors have addressed the concerns raised in first version of article and even improved the manuscript by incorporating CRISPR-Cas9 screening data analysis in the software tool. So in my opinion manuscript is now ready for indexation.

I have read this submission. I believe that I have an appropriate level of expertise to confirm that it is of an acceptable scientific standard.

# Peer review: Referee response for version 1

**DOI:** 10.5256/f1000research.4204.r4548

**Published:** 2014-05-12

**Authors:** Sumit Deswal

**Affiliations:** 1 Department of Molecular Immunology, The Research Institute of Molecular Pathology, Vienna, Austria

The authors introduce the multiplex shRNA screening approach as commonly used currently to find new drug targets or other similar applications. They also provide a literature overview on the relevant bioinformatics tools used in this context. With rapid improvements in the shRNA technology, data analysis methods for this are high in demand. The manuscript provides a good framework for analysis of data on pooled shRNA screens. There is a strong need to have such user friendly, open-source programs dedicated to analysis of shRNA screens that can easily be used by experimental biologists. The manuscript provides a coherent platform that nicely incorporates analytic tools for the current requirements of the RNAi or other similar functional genetic screens. As part of the review process, we have analyzed our own data using the software and found it fully functional and very easy to use. Based on our experience, we recommend the manuscript for indexation.

I have read this submission. I believe that I have an appropriate level of expertise to confirm that it is of an acceptable scientific standard.

# Peer review: Referee response for version 1

**DOI:** 10.5256/f1000research.4204.r4552

**Published:** 2014-05-08

**Authors:** Ross Lazarus

**Affiliations:** 1 Computational Biology, Baker IDI Heart and Diabetes Institute, Melbourne, Vic., Australia

This is a well written paper describing a potentially useful application for the analysis shRNA-seq screening data. It includes a brief description of this relatively novel technique and some competing methods as well as a description of the method used in this implementation. The authors are to be commended for providing a wrapper for Galaxy which will make it very easy for biologists to access the method in a reproducible analysis environment and this seems likely to improve the real availability and eventual impact of their work.

I have read this submission. I believe that I have an appropriate level of expertise to confirm that it is of an acceptable scientific standard.

# Peer review: Referee response for version 1

**DOI:** 10.5256/f1000research.4204.r4546

**Published:** 2014-05-01

**Authors:** James W. MacDonald

**Affiliations:** 1 Department of Environmental and Occupational Health Sciences, University of Washington, Seattle, WA, USA

This is a useful manuscript, detailing the author's extensions of existing functionality within the Bioconductor edgeR package to include shRNA screen data.

The manuscript itself provides an overview of shRNA screens, competing analysis pipelines, and the methods available in edgeR. In addition, the authors provide a link to a vignette that gives example analyses of four different shRNA experiments that vary in depth of sequencing and complexity, along with the data so potential users can recapitulate the analyses provided, before making an attempt to analyze their own data.

I have read this submission. I believe that I have an appropriate level of expertise to confirm that it is of an acceptable scientific standard.
